# Biosynthesis and biocompatibility evaluation of zinc oxide nanoparticles prepared using *Priestia megaterium* bacteria

**DOI:** 10.1038/s41598-024-54460-8

**Published:** 2024-02-20

**Authors:** Mona A. Ashour, Basma T. Abd-Elhalim

**Affiliations:** Department of Agricultural Microbiology, Faculty of Agriculture, Ain Shams University, Shubra El-Khaimah, Cairo, 11241 Egypt

**Keywords:** Cytotoxicity, Half-maximal inhibitory concentration (IC_50_), Human bone marrow cell line, Human skin melanoma, *Priestia megaterium*, Zinc oxide nanoparticles, Nanobiotechnology, Biotechnology, Nanoscience and technology

## Abstract

The current study aimed to find an effective, simple, ecological, and nontoxic method for bacterial green synthesis of zinc oxide nanoparticles (ZnONPs) using the bacterial strain* Priestia megaterium* BASMA 2022 (OP572246). The biosynthesis was confirmed by the change in color of the cell-free supernatant added to the zinc nitrate from yellow to pale brown. The *Priestia megaterium* zinc oxide nanoparticles (Pm/ZnONPs) were characterized using UV–Vis spectroscopy, high-resolution transmission electron microscopy (HR-TEM), energy-dispersive X-ray spectroscopy (EDX), Fourier transform infrared spectroscopy (FTIR), and zeta potential. The Pm/ZnONPs characterization showed that they have a size ranging between 5.77 and 13.9 nm with a semi-sphere shape that is coated with a protein-carbohydrate complex. An EDX analysis of the Pm/ZnONPs revealed the presence of the shield matrix, which was composed of carbon, nitrogen, oxygen, chlorine, potassium, sodium, aluminum, sulfur, and zinc. The results of the FTIR analysis showed that the reduction and stabilization of the zinc salt solution were caused by the presence of O–H alcohols and phenols, O=C=O stretching of carbon dioxide, N=C=S stretching of isothiocyanate, and N–H bending of amine functional groups. The produced ZnONPs had good stability with a charge of − 16.2 mV, as evidenced by zeta potential analysis. The MTT assay revealed IC_50_ values of 8.42% and 200%, respectively, for the human A375 skin melanoma and human bone marrow 2M-302 cell lines. These findings revealed that the obtained Pm/ZnONPs have the biocompatibility to be applied in the pharmaceutical and biomedical sectors.

## Introduction

It is well known that supplemental minerals and their nanoform derivatives are safe and biocompatible to consume by humans and that they have broad-spectrum antimicrobial activity at low doses^1^. Because it may be possible to create effective bio-nano materials with precise biological activities, zinc and its oxide nanoparticle substances are gaining increasing attention^2,3^. Comparing the green amalgamation of NPs to physical and chemical synthesis approaches, it has been shown that it is less hazardous, requires less work, and is environmentally benign. Nowadays, there is a significant push to innovate green biosynthesis techniques to produce a high yield of biocompatible nanoparticles. ZnO particles are generally utilized in testing biological methods and analyzing them in many biomedical applications. ZnO is one of the most significant semiconducting resources due to its diverse applications and exceptional properties. ZnO is a substance that may be found in nature and is biocompatible, which is necessary, especially for uses in the biomedical industry^3,4^. There is now a significant quantity of knowledge available thanks to recent studies on biological nanoparticles. Reductants, solvents, and ligating agents for nanoparticle formation have all been thoroughly studied to achieve the biological nanoscience goals mentioned above^5^. Natural, environmentally friendly nanoparticle amalgamation techniques avoid using any dangerous chemicals throughout the amalgamation process. As a result, these developed methods that rely on naturally occurring biomaterials offer a choice in how to get the nanoparticles that the industry needs. The synthesis of ZnONPs with inorganic complexes utilizing naturally occurring ligating agents derived from biological resources is one of the main areas of interest for the research of biological nanostructures^6^. In addition to the qualities already described, these ZnONPs that have been combined with bacterial extracts and supernatants are also known to possess beneficial antibacterial, anti-inflammatory, and anticancer qualities^7^. Extracellular biomass-free production was investigated for many metal nanoparticle investigations of many bacterial strains, such as *Bacillus* sp., *Pseudomonas* sp., and *Streptomyces* sp.^8^. The intermediate stage of research on the biological generation of metallic nanoparticles is being dominated by bacteria due to their ability and tolerance for metal bioaccumulation. Organic acids, phenolic derivatives, polyphenols, dihydroxy benzene, The reduction may be explained by the existence of numerous aromatic rings, OH groups, and groups., bioformation, and capping of many nanoparticles. The goal of the current study was to determine the biocompatibility and cytotoxicity of ZnONPs, which could be used in many future biotechnological fields. The bacterial biosynthesis of ZnONPs was carried out using a local bacterial strain as a trial to overcome the hazards caused by the physical and chemical approaches.

## Material and methods

### Chemicals and reagents

Zinc sulfate (ZnSO_4__·_7H_2_O) was purchased from Sigma, Aldrich, Germany, and was used for zinc solution preparation. Nutrient broth and agar medium were purchased from Oxoid, UK. 3-(4, 5-dimethyIthiazol-2-y1)-2-5-diphenyl tetrazolium bromide (MTT) was obtained from Merck KGaA (Darmstadt, Germany). 10% fetal bovine serum (FBS), 2 mM L-glutamine solution, 100 units/ml penicillin G sodium, 100 units/ml streptomycin sulfate, and 250 g/ml amphotericin B were obtained from Lonza, Basel, Switzerland. All chemicals were analytical grades.

### Bacteria strain

*Priestia megaterium* BASMA 2022 (OP572246) strain was collected from the Microbiology Department, Faculty of Agriculture, Ain Shams University. The strain was maintained and subcultured periodically in a nutrient broth medium and preserved at 4 °C.

## Culture filtrate preparation

The culture filtrate was prepared by inoculating a loop of freshly prepared microbial culture into the sterilized nutrient broth medium^9^ in a 100-ml flask. The inoculated flask was then incubated for 24 h at 30 °C in an orbital shaker incubator (Shin Saeng; South Korea) at 150rpm, and O.D. was observed at 620 nm^10^. One ml of the inoculum contained 2.55 × 10^6 ^CFU/ml.

### Biosynthesis of the ZnONPs using* P. megaterium* (Pm/ZnONPs)

The *P. megaterium* zinc oxide nanoparticles were synthesized following the procedure described by Kasana et al^11^. After 12 h of inoculum incubation, 50 ml of sterilized 1 mM ZnSO_4__·_7H_2_O solution was added under aseptic conditions, then re-incubated (150 rpm) at 30 °C for 24 h. The biosynthesis of Pm/ZnONPs was indicated by changing the color of the reaction mixture from pale yellow to pale brown. After that, the promising nanoparticle-supernatant mixture solution was centrifuged using the SIGMA 2–16 P, USA centrifuge at 10,000rpm for 10 min. The bacterial pellets were discarded, whereas the cell-free supernatant was collected separately to investigate the extracellular biosynthesized Pm/ZnONPs.

### *P. megaterium* zinc oxide nanoparticle characterization

The preliminary investigation for Pm/ZnONPs formation was detected by a color change of *the P. megaterium-*1 mM ZnSO_4__·_7H_2_O reaction mixture from pale-yellow to dark. The investigation of Pm/ZnONPs biosynthesis was detected through UV–vis spectroscopy (UNICO UV-2100, China) at a wavelength of 400–700 nm^12^. HR-TEM (JEOL JEM-2100, Japan) was utilized to investigate the shape regulation and poly-dispersity of Pm/ZnONPs using an amorphous carbon-coated copper grid loaded with an aliquot of Pm/ZnONPs suspension, dried, and analyzed with a 20.0 kV accelerating voltage. The TEM images were captured with an image resolution of 512 by 442 and an image pixel size of 0.04 μm^12,13^. The polydispersity of the produced nanoparticles was expressed as PDI by estimating the average size of nanoparticles and the standard deviation of synthesized nanoparticles^13^. PDI was calculated as follows^:^

1 $$\mathrm{PDI }=\upsigma /{\text{RAvg}}$$

whereas PDI = polydispersity index; σ = standard deviation of nanoparticle size; and RAvg = nanoparticle average size.

The subsequent investigations, including concentration estimation, were carried out using an atomic absorption spectrophotometer (Perkin Elmer A Analyst 100; Canada), whereas Fourier transform infrared spectroscopy (FTIR) (Perkin Elmer, 400-FTIR; USA) was used in the wavenumber range 4000–400 cm^−1^, with a resolution of 4 cm^−1^ and a refractive index of 2.4. FTIR was used for the identification of the functional groups in the *P. megaterium* supernatant responsible for reducing the ZnSO_4__·_7H_2_O, according to Izzi et al^8^. Elemental content and surface images of Pm/ZnONPs^8,12, 13^ were detected using SEM–EDX (Quanta FEG 250, FEI Company, Hillsboro, Oregon, USA) at The Desert Research Center in Egypt (EDRC), Cairo, Egypt.

### Cytotoxicity activity assay of Pm/ZnONPs

The cytotoxicity of the biosynthesized Pm/ZnONPs on cell viability was assessed at Creative Egyptian Biotechnologists (CEB), Cairo, Egypt using an MTT assay. For calibration before the MTT assay, the suspension cells in the 96-well plate were spined at 2500rpm at 4 °C for 5 min in a microplate-compatible centrifuge (CAPPRondo Microplate Centrifuge, Germany) and carefully aspirated. Ensure that the same volume of existing media is present for each sample. Then add 50 µl of serum-free medium and 50 µl of MTT solution into each well^14^. Incubate the plate at 37 °C for 3 h. Human A375 skin melanoma and human bone marrow 2M-302 cell lines were routinely cultured in RPMI. Fetal bovine serum (FBS) at 10%, 2 mM L-glutamine, 100 units/ml penicillin G sodium, 100 units/ml streptomycin sulfate, and 250 g/ml amphotericin B are added as supplements. Cells were kept in humidified air with 5% CO_2_ at 37 °C when sub-confluent. After trypsin/EDTA treatment at 37 °C, monolayer cells were collected for subculturing. When confluence had reached 75%, cells were utilized. Another aliquot of 100 μl of medium containing different doses of medicines was used to treat the cells. After 48 h of drug exposure, medium was discarded, and MTT solution (20 μl of 1 mg/ml stock solution) was added to 100 μl of phosphate buffer solution (PBS) in each well and incubated at 37 °C for 4 h. Then the formed formazan crystals were dissolved in 100 μl of absolute DMSO. The absorbance of formazan solutions was measured at λmax 570 nm using an ELISA plate reader (FLUOstar OPTIMA, BMG LABTECH GmbH, Ortenberg, Germany).

### Statistical analysis

Cytotoxicity and IC_50_ data are reported as mean ± SD (n = 3) using Graph Pad Prism 8.4.1 (GraphPad Software, San Diego, CA, www.graphpad.com), and the interaction was found to be significant at *P* < 0.05.

### Ethical approval

This article does not contain any studies with human participants or animals performed by any of the authors.

## Results

### Biosynthesis of Pm/ZnONPs

The biosynthesis of Pm/ZnONPs was detected gradually by the change in color from a pale yellow color (before the reduction of Zn) to a pale brownish solution (after the reduction of Zn), indicating the formation of ZnO nanoparticles.

### Characterization of the biosynthesized Pm/ZnONPs

#### UV–Vis spectroscopic analysis of Pm/ZnONPs

**Figure 1:**
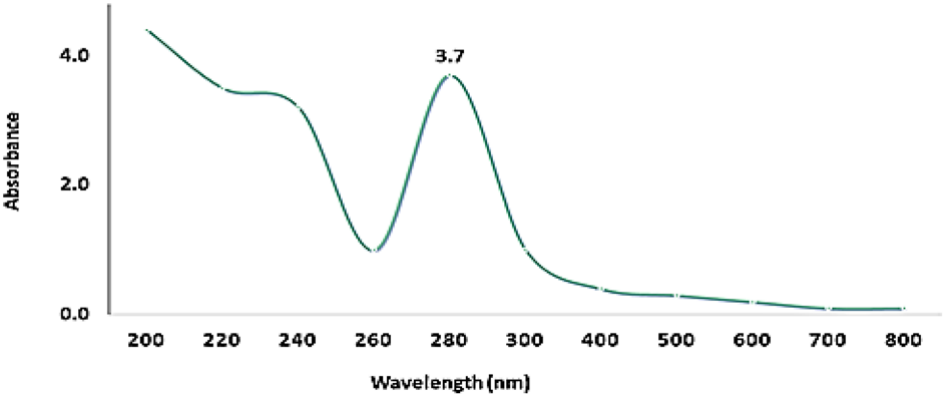
UV–Vis spectrum of green synthesized Pm/ZnONPs.

UV–Vis spectroscopy (Fig. 1) of Pm/ZnONPs indicated a high absorption spectrum between 200 and 800 nm, with maximum absorption at 280 nm and a surface plasmon resonance (SPR) peak of 3.7.

#### High-resolution transmission electron microscope (HR-TEM) analysis of the biosynthesized Pm/ZnONPs

**Figure 2:**
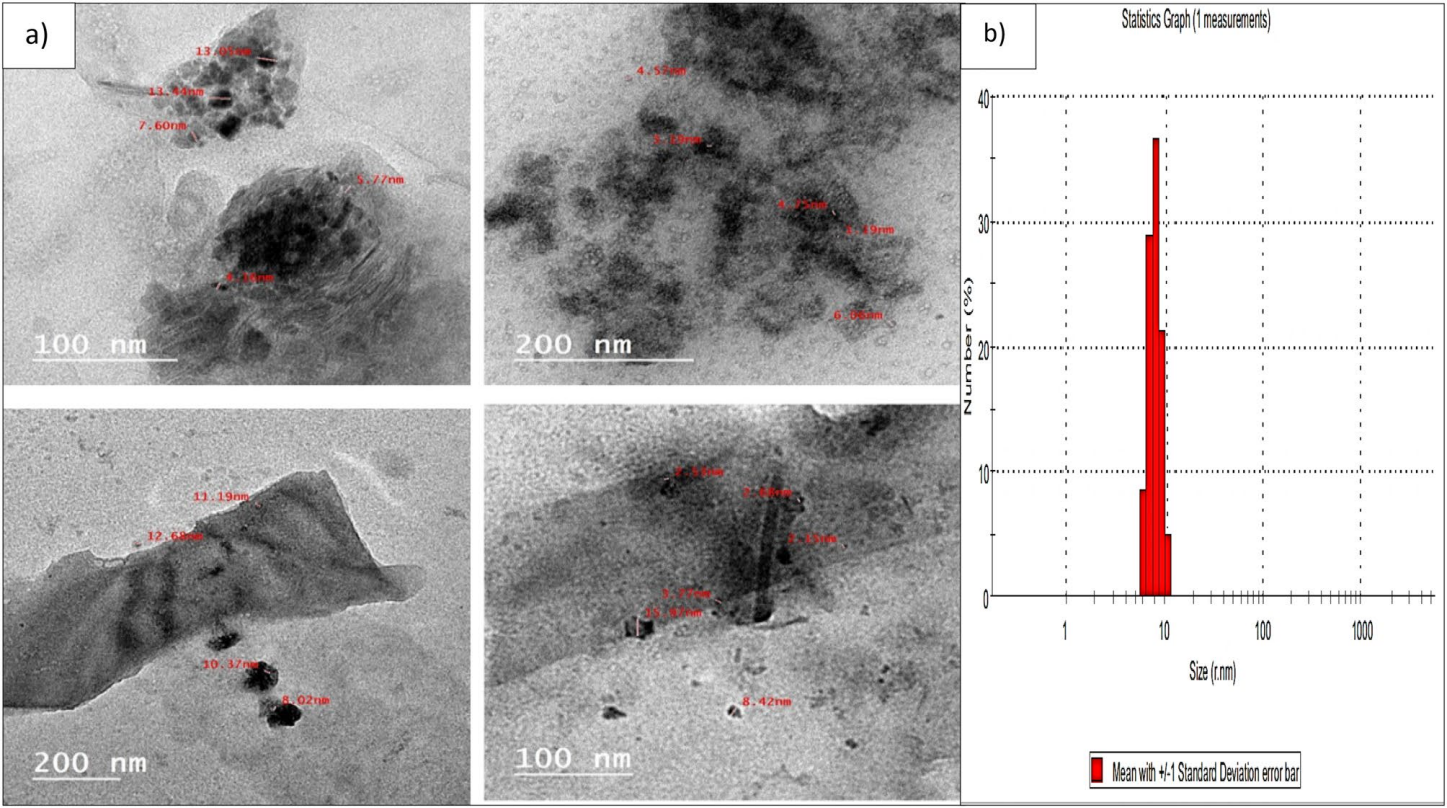
High-resolution transmission electron microscopy (HR-TEM) image of the biosynthesized Pm/ZnONPs, (**a**) Morphology and size of Pm/ZnONPs, (**b**) Mean standard division of Pm/ZnONPs.

The morphology and size of the biosynthesized Pm/ZnONPs were observed by HR-TEM (Fig. 2). The particles were semi-spheres and well-despised, with a size range of 5.77–13.9 nm. The polydispersed nature of particles was confirmed with a PDI value of 0.15.

#### Energy-dispersive X-ray spectroscopy (EDX) analysis of the biosynthesized Pm/ZnONPs

**Figure 3:**
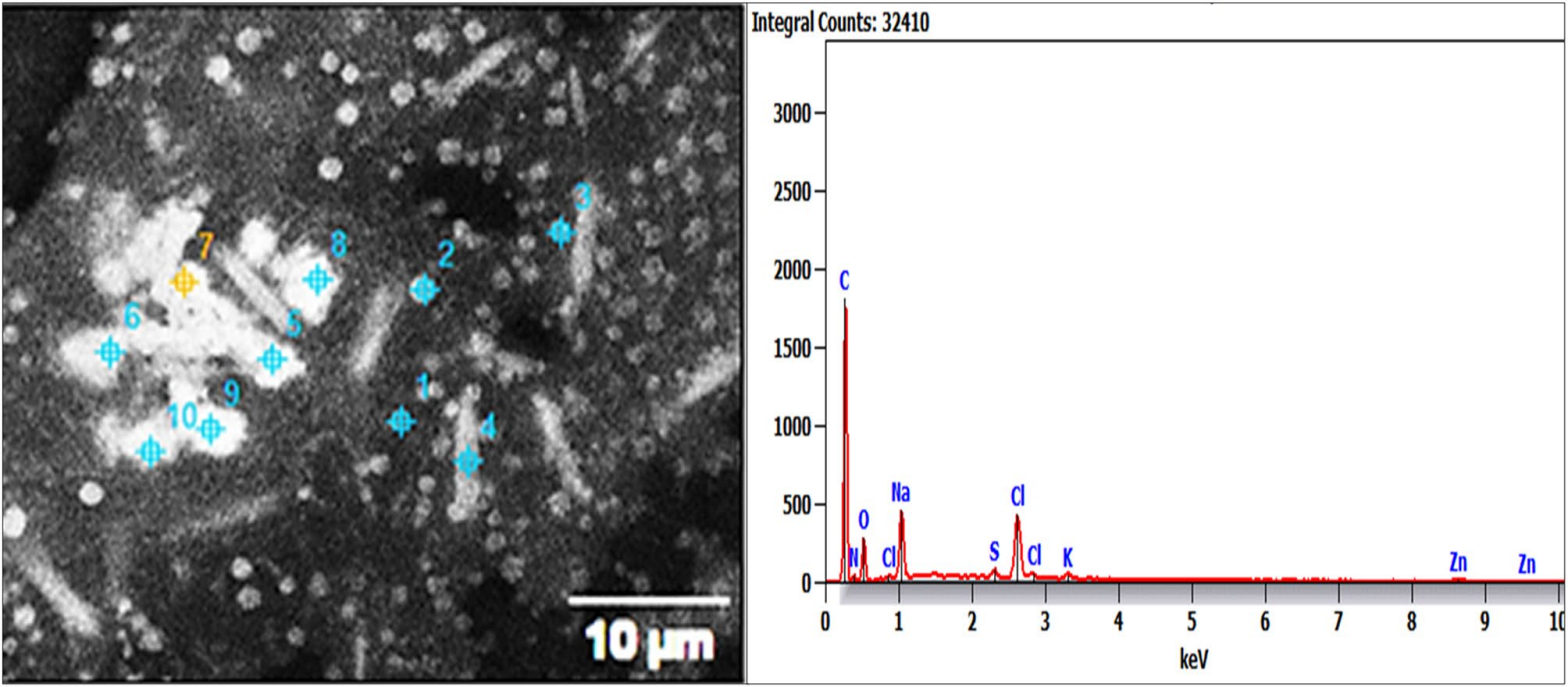
Energy-dispersive X-ray (EDX) spectroscopy of the biosynthesized Pm/ZnONPs.

Energy-dispersive X-ray (EDX) investigation of Pm/ZnONPs confirmed the presence of the shield matrix, which consisted of carbon, nitrogen, oxygen, chlorine, potassium, sodium, aluminum, sulfur, and zinc, attached to Pm/ZnONPs with percentages of 40.2, 15.5, 26.3, 5.7, 0.6, 1.4, 7.3, 1.2, and 1.8%, respectively. This indicates active biomolecule formation that coated the ZnONPs (Fig. 3).

### Fourier transformation infrared (FTIR) characterization of the biosynthesized Pm/ZnONPs

**Figure 4:**
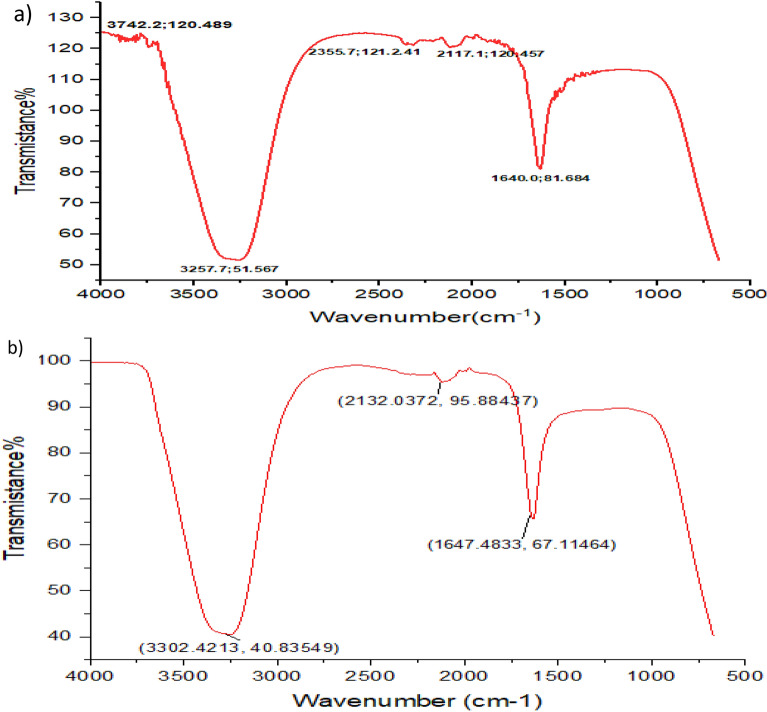
Fourier transmission infrared spectroscopy (FTIR) spectrum of, (**a**) the biosynthesized Pm/ZnONPs. (**b**) *P. megaterium* culture supernatant (control).

The Fourier transformation infrared (FTIR) characterization analysis of the biosynthesized Pm/ZnONPs is illustrated in Fig. 4. The infrared spectrum shows five mean independent peaks at 3742.24, 3257.69, 2355.67, 2177.12, and 1640.02 cm^− 1^. This spectrum clarifies the presence of O–H in alcohols and phenols, O=C=O stretching of carbon dioxide, N=C=S stretching of isothiocyanate, and N–H bending of amine.

### Zeta potential determination of the biosynthesized Pm/ZnONPs

**Figure 5:**
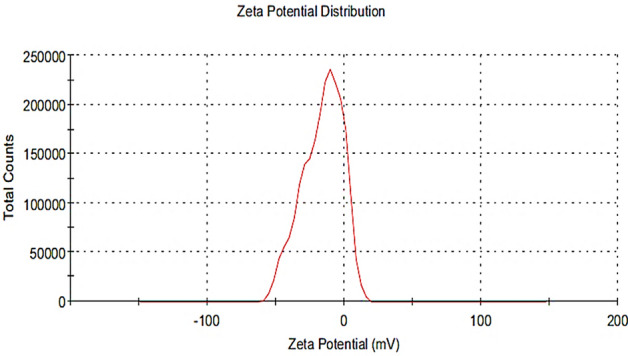
Zeta potential analysis of Pm/ZnONPs showed a -16.2 mV charge.

As shown in Fig. 5, Pm/ZnONPs have a zeta potential of − 16.2 mV, which can be attributed to the nonionic character of the capping molecules in the *P. megaterium* culture supernatant. This negative charge indicates the good stability of the biosynthesized ZnONPs.

### Assessment of the biosynthesized Pm/ZnONPs cytotoxicity

**Figure 6:**
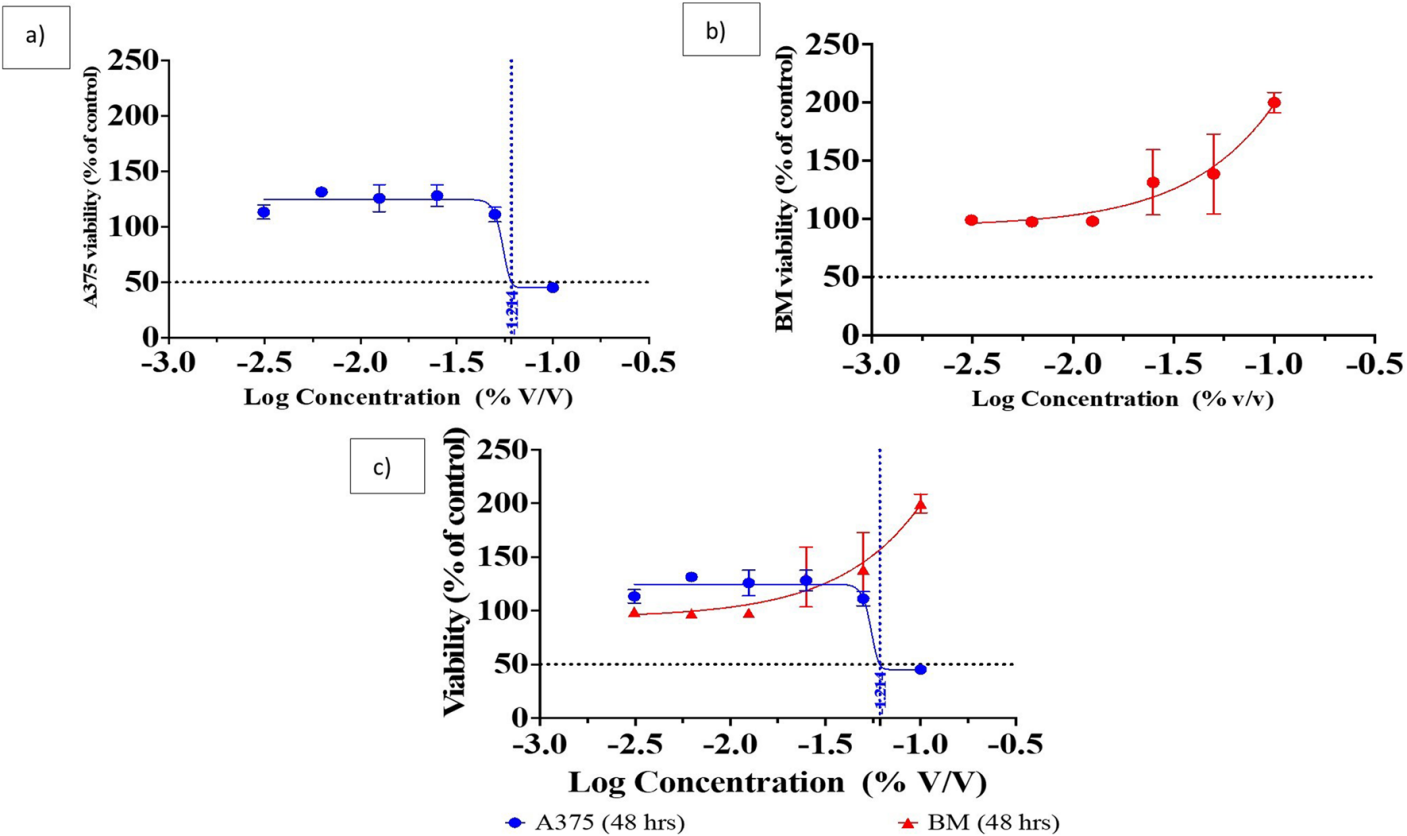
The dose curve of the various biosynthesized Pm/ZnONPs concentrations and IC_50_ doses using the human A375 skin melanoma and human bone marrow 2M-302 cell lines. (**a**) Human A375 skin melanoma; (**b**) Bone marrow 2M-302 cell lines; and (**c**) Human A375 skin melanoma versus Bone marrow 2M-302 cell line.

The MTT assay revealed different effects of each different sample on the cell lines. The Pm/ZnONPs sample was toxic to the skin A375 cell line with an IC_50_ equal to 8.42% v/v. The cell viability (%) was 47.36, 116.76, 132.92, 134.01, 133.57, and 115.67% for Pm/ZnONPs concentrations of 10, 5, 2.5, 1.25, 0.625, and 0.312 μg/ml, respectively, with an IC_50_ dose of > 6.1% Pm/ZnONPs as presented in Fig. 6a. While the bone marrow cells showed increased proliferation up to 200% in a dose-dependent manner, The cell viability (%) was 208.76, 158.04, 148.07, 94.72, 95.18, and 100.12% for Pm/ZnONPs concentrations of 10, 5, 2.5, 1.25, 0.625, and 0.312 μg/ml, respectively, as presented in Fig. 6a, b.

## Discussion

As known, ZnONPs have been listed as “Generally Recognized as Safe” (GRAS) by the US Food and Drug Administration (FDA 21CFR182.8991) due to their non-toxic properties^15,16^. In the present investigation, bio-augmented zinc oxide nanoparticles (ZnONPs) were prepared from *P. megaterium* BASMA 2022 (OP572246) bacteria extracellular supernatant^17^. Biological synthesis using microbes offers an advantage over plants since microbes are easily reproduced. The synthesis of metal and metal oxide NPs depends on the ability of microbes to tolerate heavy metals. Moreover, it is well known that high metal stress may affect various microbial activities^18–20^. Under stressful environments, the microbes tend to reduce metals to ions. As such, this demonstrates their capability to act as a natural nano factory^21,22^. Generally, microbes that inhabit rich metals exhibit metal resistance due to chelation by extracellular proteins^17^. Many bacteria were reported for extracellular biosynthesis of ZnONPs, such as *Bacillus licheniformis* MTCC9555, *Bacillus megaterium* (NCIM2326), *Lactobacillus paracasei* LB3, and *Lactobacillus sporogenes*^22–25^.

The biological synthesis of metal and its oxide NPs requires metal precursors, which are usually supplied in the form of soluble salts and precipitated in the suspension containing microbial cells and/or biological compound extracts from the microbe’s culture growth. The synthesis reaction is usually completed within minutes or hours, depending on the culture conditions, which results in white deposition in the bottom flasks or changes in the color of suspensions^16,21^. This observation in color change was confirmed by many previous studies^4,5, 20, 26, 27^.

The room-temperature UV–Vis absorbance spectrum was identified as the surface plasmon resonance (SPR) that is a characteristic of metal nanoparticles and their oxides^23,24^. The confirmation investigation of Pm/ZnONPs formation was using UV–Vis spectroscopy at an absorption spectrum between 200 and 800 nm with maximum absorption at 280 nm and SPR peak of 3.7. Biosynthesis of Pm/ZnONPs was detected gradually by the change in color from a pale yellow color to a brownish solution, indicating the formation of ZnO nanoparticles due to the excitation of nanoparticles’ surface plasmon resonance (SPR). On the other hand, it was reported that the UV–vis spectra results indicated a strong and broad peak at 250 and 374 nm, implying the successful formation of ZnONPs^5,17, 28^.

HR-TEM investigation of the biosynthesized Pm/ZnONPs indicated the average diameter of the biosynthesized Pm/ZnONPs was 5.77–13.9 nm of semi-spheres and well-despised nanoparticles. While in other reports it was reported to be lower than in the findings of Selvarajan and Mohanasrinivasan^29^ demonstrated a spherical shape ZNO–NP synthesis using *Lactobacillus plantarum* VITES07 with size ranging from 7 to 19 nm and *B. licheniformis* MTCC9555 with size at 250 nm^23^. In the study of Król et al^24^, ZnONPs mediated *L. paracasei* LB3 with a larger size of 1179 nm. The spherical and semi-sphere shapes of ZnONPs were observed in many reports^1,4, 27, 30, 31^.

The EDX investigation of Pm/ZnONPs confirmed the presence of a strong shield matrix coat that consisted of C, N, O, Cl, K, and Na that attached to ZnONPs with 40.2, 15.5, 26.3, 5.7, 0.6, 1.4, 7.3, 1.2, and 1.8%, respectively. This indicates active biomolecule formation that coated the ZnONPs. These elements indicate that the presence of various enzymes, proteins, and other biomolecules from *P. megaterium* cell-free supernatant plays a vital role in the reduction process of Zn metal solution. These multiple organic components secreted in the suspension or growth medium are attributed to the formation of multiple sizes and shapes of mono- and polydispersed NPs^31,32^. The capping effect of the accumulated active compounds on the metal nanoparticle core is responsible for the reduction, stability, and capping of the nanoparticles, as described by Mohamed^31^.

The FTIR characterization analysis of the biosynthesized Pm/ZnONPs showed mean five independent peaks at (3742.24 and 3257.69), 2355.67, 2177.12, and 1640.02 cm^−1^. This spectrum clarifies the presence of O–H in alcohols and phenols, O=C=O stretching of carbon dioxide, N=C=S stretching of isothiocyanate, and N–H bending of amine. Velmurugan et al^33^. findings revealed the presence of protein and amide, with one and two peaks at 1100, 1400, 1650, 2900, and 3000 cm^−1^, respectively. Nevertheless, there was no protein signal detected in the zinc crystal produced by the dead biomass of *Fusarium* spp.. Similarly^34^, evaluated the chemical composition of the ligands capping the NPs. The FTIR results demonstrated two absorption bands at 1650 and 1566 cm^−1^, which indicated the typical amide absorptions of protein molecules. These findings were in line with the findings obtained by^35^, as biomolecules were identified as the molecules that had the ability for biosynthesized NP capping and stabilization. It was found that the N–H peak appeared at 1640.02 cm^−1^ and covered amine groups and nitro compound bonds that identify the bounds of protein groups responsible for biosynthesis and between the biosynthesized nanoparticles as stabilizing caps attached to proteins and amino acid residues. Numerous investigations have also suggested that nitrate reductase is involved in extracellular production, which results in the reduction of metal ions into metal NPs^24,36–38^.

The extracellular synthesis route entails either enzyme-mediated synthesis occurring on the cell membrane or the release of the enzyme as an extracellular enzyme into the growth medium. Nitrate reductase is an enzyme in the nitrogen cycle that catalyzes the conversion of nitrate to nitrite. For instance, NADH-dependent reductase, which serves as an electron carrier, transferred an electron from NADH to begin the bioreduction of Zn^2+^^24,39^. As a result, Zn^2+^ attracted electrons and transformed them into Zn^0^ and ZnONPs were then created as a result of this^35^. The Pm/ZnONPs have a zeta potential of − 16.2 mV, which can be attributed to the nonionic character of the capping molecules in the *P. megaterium* culture supernatant. And indicated that the nanoparticles synthesized were highly stable. It scored a zeta potential value of − 33.4 mV with *Serratia nematodiphila*^40^ and *Pseudomonas aeruginosa*^41^ with − 18.0 mV.

As reported by^17,42^ despite ZnONPs potential use as a feed supplement, it also tends to cause adverse effects on animals and human cells. However, the toxicological hazards of ZnONPs remain controversial because, while a few studies have reported ZnONPs to have therapeutic benefits, other studies have reported their toxicity to living organisms. The MTT assay of cytotoxicity assessment revealed various effects on the human A375 skin melanoma cell line, with an IC_50_ of 8.42% for Pm/ZnONPs. While the human bone marrow 2M-302 cell line showed increased proliferation up to 200% in a dose-dependent manner, studies have suggested that the toxicity effects of ZnONPs are dependent on their dose (concentration)^42^, morphology and composition^43^ and size^44^. As reported, the smaller size of NPs ranging between 3 and 6 nm is more easily cleared out of the kidneys compared to bigger NPs with a size near 30 nm, which remain and accumulate in the liver. In addition^45–47^ reported that larger NPs also tend to stay longer in the kidneys and skin due to the slower excretion mechanisms of glomerular filtration, and this long-term retention can lead to organ toxicity. In addition, different morphologies of NPs also contribute to the toxicity effects, regardless of their specific surface area. investigated the cytotoxicity effects of ZnONPs with different morphologies, such as nonuplets, nanorods, nanosheets, and nanoflowers, on malignant human T98G gliomas and fibroblast cells^48^. Nanorods demonstrated higher cytotoxicity and inhibitory effects on normal and tumor cells due to a larger effective surface area that potentially induces higher oxidative stress on cells.

Due to the circumstances of the chemical reaction in the usual approach, it was also noted that the chemically and physically manufactured ZnONPs might be one of the potential sources of the inherent toxicity of NPs^47^. It has been hypothesized that ZnONPs’ harmful effects result from their ability to readily penetrate cells, attach to membranes, or release Zn^2+^, which causes oxidative stress-mediated DNA damage and lipid peroxidation, all of which lead to apoptosis. Several studies have reported that high doses of ZnONPs supplementation could lead to toxicity^47,49–52^. Oral administration of ZnONPs (20%) in lambs caused toxicity effects, which included increased levels of blood urea nitrogen (BUN) and creatinine, indicating renal dysfunction^51^. Also, results of an in vivo experiment conducted by Wang et al^53^., showed that by reducing body weight and increasing the relative weight of the pancreas, brain, and lung in mice, high dosages of ZnO-NP supplementation at 50% resulted in toxicity. In addition, zinc buildup was seen in the bones, kidney, liver, and pancreas. Meanwhile, long-term exposure to ZnONPs at 5% only showed minimal toxicity. Furthermore, in the histopathological examination, a high concentration of oral administration of ZnONPs at 4% induced focal hemorrhages and necrosis on the liver and heart tissue of Wistar rats, which were caused by oxidative stress^53,54^. Also, it was discovered that the surface-bound active compounds on the surface of NPs play a crucial role in their biological interactions.

As reported previously, coatings for the surface of ZnONPs were effective in reducing their cytotoxicity effect on epithelial cells by restricting the dissociation of ZnONPs to Zn^2+^^55,56^. On the other hand, the current findings demonstrate that Pm/ZnONPs stimulate the synthesis of bone marrow cells and may be used to treat bone marrow production deficiencies. Deylam et al^57^. reported that ZnONPs with average sizes of 10–30 and 35–45 nm on bone marrow and mesenchymal stem cells (MSCs) were found to be safe at concentrations of 5 and 10 µg/ml. As the cell-cycle analysis indicated, they upregulate the aging-related genes NF–kB and p53 and downregulate the anti-aging gene Nanog. Wang et al^58^, discovered that zinc-whitlockite ((ZnWH)/G/H) nanoparticles exhibited interconnected pore structures, outstanding mechanical characteristics, and tunable swelling ratios. The high quantities of alkaline phosphatase (ALP), osteocalcin (OCN), and osteopontin that are released by human bone marrow mesenchymal stem cells (hBMSCs) can induce osteogenic development in addition to their good biocompatibility (OPN). The ZnWH scaffold dramatically sped up the process of bone restoration after 12 weeks of therapy in the rabbit femoral defect model, making it a viable choice for bone regeneration. In summary, the toxic effects of ZnONPs are caused by their dosage, size, and shape; thus, the use of ZnONPs in many applications should be restricted to a specific minimum concentration to avoid their toxic effects. Moreover, for improved safety of Pm/ZnONPs, microbe-mediated synthesis should be considered in NP production due to its biocompatibility as well as controllable NPs size and shape, which can be achieved through the optimization process.

## Conclusion

The study aimed to develop a simple, eco-friendly, and non-toxic method for bacterial green synthesis of zinc oxide nanoparticles using *P. megaterium* BASMA 2022 (OP572246). The resulting Pm/ZnONPs were characterized using various techniques, including UV–Vis spectroscopy, HR-TEM, EDX, FTIR, and zeta potential. The Pm/ZnONPs had a semi-sphere shape and good stability, with IC_50_ values of 8.42 and 200% for human A375 skin melanoma and human bone marrow 2M-302 cell lines, respectively. These findings could help in future applications in various sectors such as agriculture, nutrition, pharmaceuticals, and biomedical research.

## Data Availability

The datasets generated during the current study are available in the [NCBI] repository, https://www.ncbi.nlm.nih.gov/nuccore/OP572246
